# Three-dimensional preservation of cellular and subcellular structures suggests 1.6 billion-year-old crown-group red algae

**DOI:** 10.1371/journal.pbio.2000735

**Published:** 2017-03-14

**Authors:** Stefan Bengtson, Therese Sallstedt, Veneta Belivanova, Martin Whitehouse

**Affiliations:** 1 Department of Palaeobiology, Swedish Museum of Natural History, Stockholm, Sweden; 2 Nordic Center for Earth Evolution (NordCEE), Odense, Denmark; Copenhagen, Denmark; Stockholm, Sweden; 3 Department of Geosciences, Swedish Museum of Natural History, Stockholm, Sweden; Massey University, New Zealand

## Abstract

The ~1.6 Ga Tirohan Dolomite of the Lower Vindhyan in central India contains phosphatized stromatolitic microbialites. We report from there uniquely well-preserved fossils interpreted as probable crown-group rhodophytes (red algae). The filamentous form *Rafatazmia chitrakootensis* n. gen, n. sp. has uniserial rows of large cells and grows through diffusely distributed septation. Each cell has a centrally suspended, conspicuous rhomboidal disk interpreted as a pyrenoid. The septa between the cells have central structures that may represent pit connections and pit plugs. Another filamentous form, *Denaricion mendax* n. gen., n. sp., has coin-like cells reminiscent of those in large sulfur-oxidizing bacteria but much more recalcitrant than the liquid-vacuole-filled cells of the latter. There are also resemblances with oscillatoriacean cyanobacteria, although cell volumes in the latter are much smaller. The wider affinities of *Denaricion* are uncertain. *Ramathallus lobatus* n. gen., n. sp. is a lobate sessile alga with pseudoparenchymatous thallus, “cell fountains,” and apical growth, suggesting florideophycean affinity. If these inferences are correct, *Rafatazmia* and *Ramathallus* represent crown-group multicellular rhodophytes, antedating the oldest previously accepted red alga in the fossil record by about 400 million years.

## Author summary

The last common ancestor of modern eukaryotes is generally believed to have lived during the Mesoproterozoic era, about 1.6 to 1 billion years ago, or possibly somewhat earlier. We studied exquisitely preserved fossil communities from ~1.6 billion-year-old sedimentary rocks in central India representing a shallow-water marine environment characterized by photosynthetic biomats. We discovered amidst extensive cyanobacterial mats a biota of filamentous and lobate organisms that share significant features with modern eukaryotic algae, more specifically red algae. The rocks mainly consist of calcium and magnesium carbonates, but the microbial mats and the fossils are preserved in calcium phosphate, letting us view the cellular and subcellular structures in three dimensions with the use of synchrotron-radiation X-ray tomographic microscopy. The most conspicuous internal objects in the cells of the filamentous forms are rhomboidal platelets that we interpret to be part of the photosynthetic machinery of red algae. The lobate forms grew as radiating globular or finger-like protrusions from a common centre. These fossils predate the previously earliest accepted red algae by about 400 million years, suggesting that eukaryotes may have a longer history than commonly assumed.

## Introduction

Multicellular eukaryotes rose to prominence around the Proterozoic–Phanerozoic transition coupled to evolving ecological interactions between megascopic autotrophs and heterotrophs. Animals in particular are thought to have had a pivotal role as—for example—predators, grazers, and filterers in expanding the food web through interactive processes not available to microbial life [1]. Fungi, the other major opisthokont multicellular clade, are important heterotrophic consumers, but their role in Phanerozoic ecosystem evolution is generally considered to be focused on the terrestrial biosphere [2–4]. The three major lineages of multicellular eukaryotes—Plantae, Fungi, and Metazoa—diversified already during the Proterozoic, but the time of eukaryote origin is under dispute (reviews by [5–9]). Estimates of the age of the last eukaryote common ancestor, LECA (the last ancestor of the eukaryote crown group), vary between extremes of about 0.8 Ga [10] and 2.3 Ga [11], though most proposed dates based on modern molecular-clock work land within the Mesoproterozoic [7,12–16] or late Palaeoproterozoic [17]. The considerable interval of uncertainty is due to difficulties in establishing the origination time of fossil taxa used for calibration as well as to other problems involved in molecular-clock dating. One major issue is the lack of robust phylogenies in the base of the eukaryotic tree [6,18–23].

The age of the first eukaryote common ancestor, FECA (the last ancestor of the eukaryote total group), is even more uncertain, an extreme proposal being early Archaean, based on evidence of methanogenesis at 3.5 Ga taken to herald the appearance of the euryarchaeotan sister group (containing methanogenic archaea) of the clade containing the Eukaryota [9]. The presence of more widespread methanogenesis in the basal archaean tree [24], however, leaves this inference without support. A more conservative minimum estimate suggests that FECA had arrived some time before 1.9 Ga based on the earliest widely acceptable eukaryote fossils [7]. However, it is in the nature of stem-group organisms (see [25] for a discussion of the stem-group and crown-group concepts in palaeontology) that initially they do not differ phenetically from representatives of their sister group, and thus, attempts to identify FECA in the fossil record are a hopeless exercise.

More pertinent is the question of when stem-group eukaryotes had acquired fossilizable features that would enable us to recognize them in the fossil record. The path from FECA to LECA may have been a fast endosymbiosis-dominated set of events [10] or a slow process marked by successive events of horizontal gene transfer [26]. To distinguish between those two scenarios, it is necessary to identify eukaryote characters correctly in the deep fossil record.

The fossil morphological record of early eukaryotes is thus of crucial importance, but most reports of pre-Cryogenian eukaryotes have at some point been challenged. The interpretation of morphologically simple fossils as early eukaryotes has mainly been based on large size, ornamentation of walls, and a complex, resistant wall ultrastructure [5,7,9,27,28]. The iconic Palaeoproterozoic tubular fossil reported as *Grypania* (which lacks the segmented structure of the Mesoproterozoic type species and may not be a true *Grypania* [29]) is generally heralded as the first plausible eukaryote in the fossil record, mainly based on its size [7,30–34], though the eukaryotic affinity has been questioned [29], with an alternative interpretation being its identification as a giant cyanobacterium [35,36]. Other early eukaryote-like fossils [37–40] are plausible as such but unconfirmed as crown-group eukaryotes.

Geochemical evidence has also been summoned in support of early eukaryotes, but even more controversially. A much-cited report of 2.7 Ga sterane biomarkers attributed to eukaryotes [41,42] has been shown to be in error, the biomarkers having been introduced as younger contaminants [43]. In spite of subsequent attempts to identify Archaean eukaryote biomarkers, all such evidence for eukaryote presence is now considered invalid, not only because of contamination issues [44] but also because the putative Archaean steranes that were taken as proxies for eukaryotic membrane sterols may also derive from noneukaryote life [45].

Establishing a minimum age for LECA implies identifying crown-group eukaryotes in the fossil record. *Bangiomorpha*, interpreted as a bangiophycean rhodophyte [46], is dated to about 1.1–1.25 Ga [47]. It represents the oldest generally accepted (though see [16,17,35,48]) fossil crown-group eukaryote. Earlier unicellular fossils interpreted as rhodophytes [49] have not found acceptance [50]. Simple acritarchs at 1.8 Ga [51] have been argued to indicate a presence of chlorophytes at that time [39], but the identification of the fossils as crown-group eukaryotes is controversial [7].

We have studied uniquely preserved fossils from the ~1.6 Ga Tirohan Dolomite at Jankikund, Chitrakoot, central India, and present here cellularly and subcellularly preserved diverse forms that show characters of rhodophytes, implying that crown-group eukaryotes, and consequently LECA, are no younger than the Palaeoproterozoic.

### Geological background

**Fig 1:**
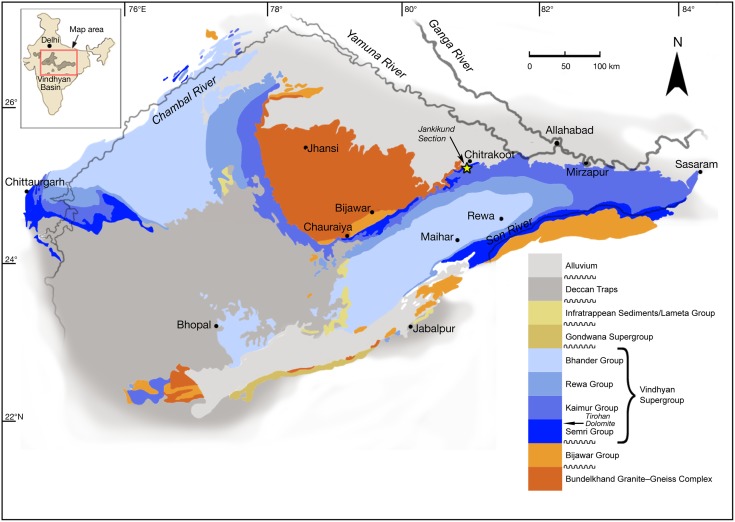
Geological map of the Vindhyan basin, central India. After Azmi et al. [55], based on several sources.

The Vindhyan Supergroup consists of a thick, unmetamorphosed sequence of sandstones, shales, and carbonate rocks, as well as volcanoclastic rocks. The ~1.6 Ga [52] Chitrakoot Formation, which includes the Tirohan Dolomite, belongs to the Lower Vindhyan (the Semri Group) and is exposed north of the Son River Valley in the Chitrakoot region, Uttar Pradesh and Madhya Pradesh, central India (Fig 1). The formation rests unconformably on the Bundelkhand Granite and is unconformably overlain by the Kaimur Sandstone [53,54].

The phosphatized stromatolitic rocks of the Tirohan Dolomite at the Paisuni River section, Jankikund, are known for their exquisite preservation of microbial fossils [52,55,56]. The unit consists of stromatolitic carbonates with phosphorite occurring as bands within and capping the stromatolites. The intercolumnar matrix contains phosphatic intraclasts. The phosphatized fossils are found both in the intercolumnar matrix and in the phosphate bands in the stromatolites. Parts of the rock are silicified. The Tirohan Dolomite was deposited in a marine shallow-subtidal to supratidal environment [53,54,57]. Deposition in a photic and oxygenic environment is demonstrated by the dominance of gas-producing cyanobacteria [52].

## Results and discussion

### Tubular forms

The tubular components of the Tirohan stromatolitic biota mostly consist of aggregating masses of entangled filaments, interpreted to represent filamentous cyanobacteria [52,56]. Furthermore, scattered among the stromatolitic layers and the intercolumnar debris are isolated tubular structures. Most of these are less than 10 μm in width and are probably sheaths of filamentous bacteria or chains of bacterial cells. Some tubes, however, are considerably larger and represent potential eukaryotic organisms. Being rare, these have not been observed in petrographic sections but have been recovered by manual selection from acid residues.

The larger tubes vary in width from 58 to 275 μm. Similar forms from the Tirohan phosphorites were initially reported and interpreted as Cambrian taxa, *Cambrotubulus decurvatus* and *Hyolithellus vladimirovae* [55]. The ensuing controversy about the age of the Lower Vindhyan and the provenance of the fossils was resolved by the demonstration that the Tirohan fossil tubes were morphologically analogous to modern filamentous algae rather than to the named Cambrian taxa and that Pb–Pb isochron analysis confirmed a late Palaeoproterozoic rather than Cambrian age of the phosphorites [52]. However, the biological nature of these enigmatic fossils has not until now been seriously investigated.

Our microtomography data clarify the internal structure of the tubular fossils, suggesting that they represent two significantly different organisms, here named *Rafatazmia chitrakootensis* n. gen., n. sp. and *Denaricion mendax* n. gen., n. sp. *Rafatazmia* ranges in width from 58 to 175 μm, *Denaricion* from 130 to 275 μm. *Rafatazmia* consists of concatenated cell-like compartments, often collapsed or distorted, resulting in buckling and folding of the exterior, whereas *Denaricion* has a solid internal structure similar to a stack of coins, upholding the integrity of the outer shape.

#### Taphonomy and diagenesis of *Rafatazmia* tubes

**Fig 2:**
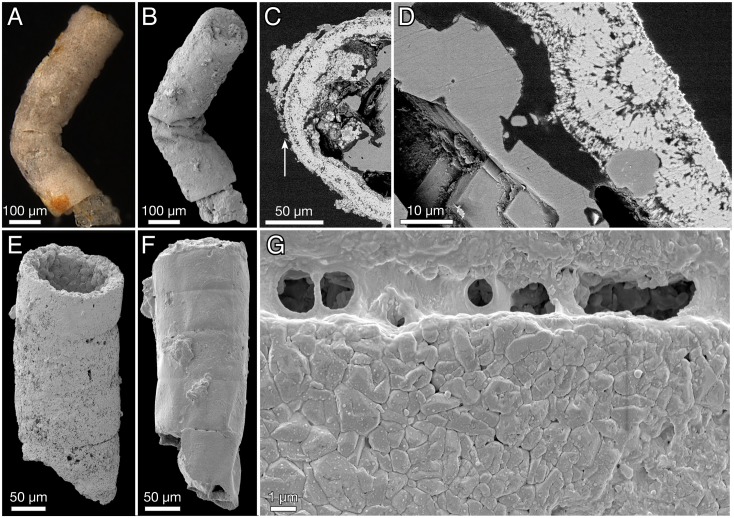
*Rafatazmia chitrakootensis* n. gen., n. sp., Scanning Electron Microscopy (SEM) images. (A–D) NRM S156424 (also figured in [52]); C and D are backscatter images of a polished transverse section. (E) NRM X5647. (F, G) NRM X5648.

**Fig 3:**
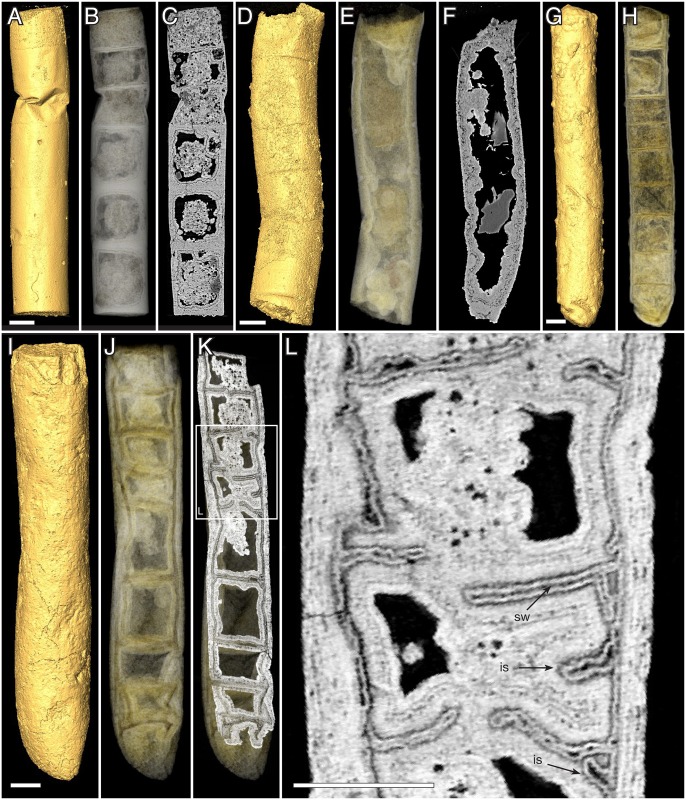
*Rafatazmia chitrakootensis* n. gen., n. sp., Synchrotron-Radiation X-ray Tomographic Microscopy (SRXTM) renderings. (A–C) NRM X5646, surface, volume, slice. (D–F) NRM X5592, surface, volume, slice. (G, H) NRM X4230, surface, volume. (I–L) NRM X4229, surface, volume, slice. Legend: sw, septal wall; is, incomplete septum. Scale bars 50 μm.

**Fig 4:**
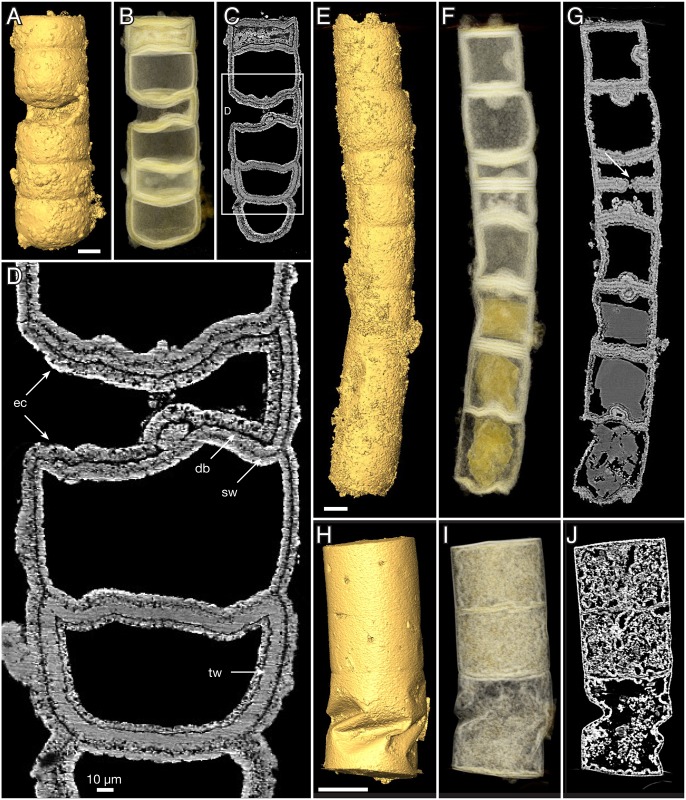
*Rafatazmia chitrakootensis* n. gen., n. sp., SRXTM renderings. (A–D) NRM X4251, surface, volume, slice. (E–G) NRM X5572, surface, volume, slice. (H–J) NRM X5562, surface, volume, slice. Legend: ec, external coating; db, diagenetic boundary; sw, septal wall; tw, thick wall. Scale bars 50 μm except where otherwise noted.

The fossilized structure of most *Rafatazmia* tubes is dominated by void-filling apatite similar to that which is common in Neoproterozoic and Cambrian phosphatized fossils [58–60]. It takes the shape of fibronormal apatite crystals forming a layered growth on the substrate (Fig 2C and 2D). Because of irregularities on the overgrown surface, the crystals frequently fan out to produce a spherulitic or botryoidal texture (Fig 2D and 2E). Growth of the layers may be directed only inwards toward the central cavities (Fig 2D) or in opposite directions starting out from both sides of the original walls (Figs 3J–3L, 4B–4D, 4F and 4G). Void-filling apatite often forms irregular structures within the compartments, occasionally even filling them completely; in the latter case, the filling can be seen to have protected the outer walls from buckling (compare the two upper compartments with the bottom one in Fig 4H–4J).

A unique specimen with one compartment open to the outside, through apparent loss of part of the outer wall, shows internal diagenetic apatite growth on both sides of the original septal wall (Fig 4D, sw) as well as an external growth layer that is continuous with a secondary layer covering the original internal coating layer (Fig 4D, ec). Taken together, these observations indicate that there are at least two generations of diagenetic void-filling apatite: the internal coating formed early and was the main agent for preserving the physical integrity of the outer and inner walls; the outer diagenetic layer, when present, is a later addition.

**Fig 5:**
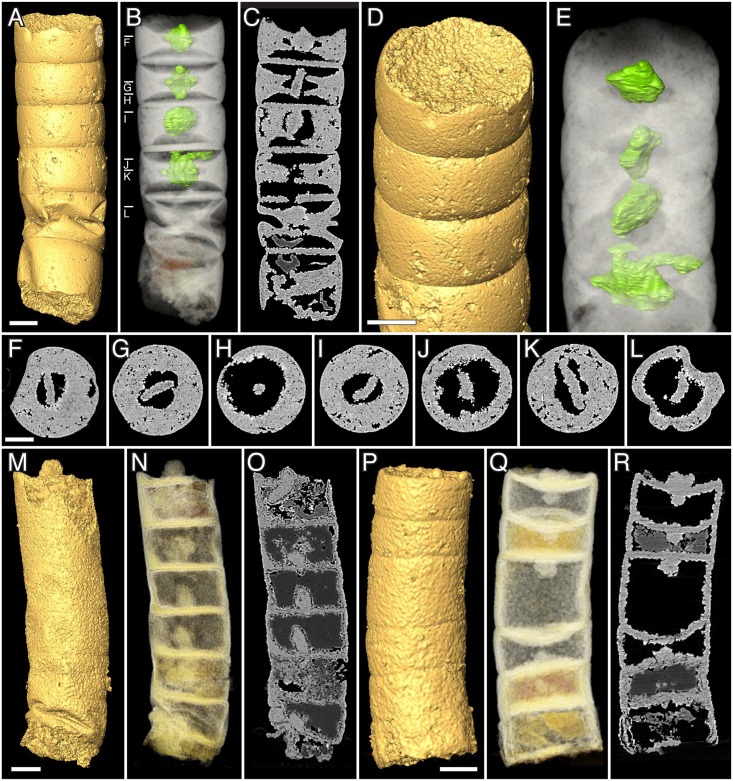
*Rafatazmia chitrakootensis* n. gen., n. sp., SRXTM renderings. (A–L) Holotype, NRM X4258. (A) Surface rendering. (B) Volume rendering with rhomboidal disks coloured for visibility. (C) Virtual slice. (D) Surface. (E) Volume. (F–L) Transverse slices (positions indicated in B). (M–O) NRM X5620, surface, volume, slice. (P–R) NRM X5574, surface, volume, slice. Scale bars 50 μm.

*Rafatazmia* tubes showing original intracellular features are preserved in microcrystalline apatite (Fig 5). This suggests that the microcrystalline apatite is an early diagenetic replacement of biological substance, whereas the layered fibronormal apatite represents later events of diagenetic incrustation. Comparable conclusions have been reached with regard to similar diagenetic fabrics in the phosphatized fossils of the Ediacaran Doushantuo Formation [59,61].

#### Morphology of *Rafatazmia* tubes

**Fig 6:**
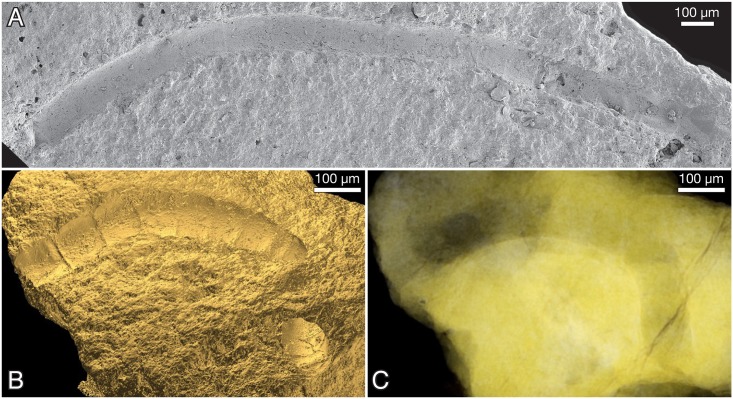
*Rafatazmia chitrakootensis* n. gen., n. sp., SEM image (A) and SRXTM renderings (B, C). A: NRM S156422 (also figured in [52]). (B, C) NRM X5544, surface, volume.

**Fig 7:**
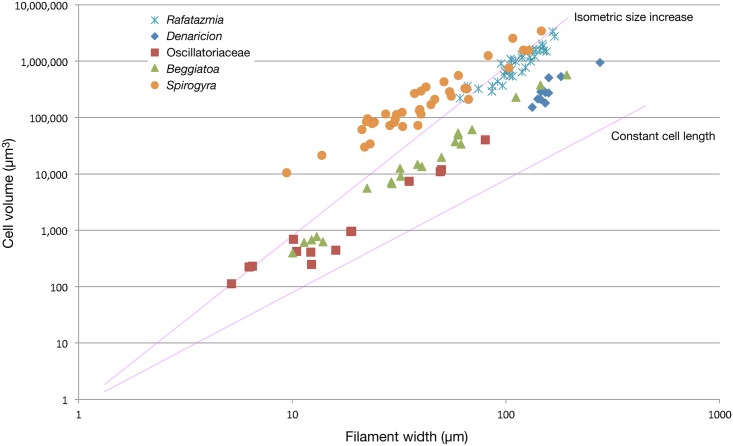
Relationships between filament width and cell volume in *Rafatazmia*, *Denaricion*, and some modern filamentous organisms. Data sources for the modern taxa: Oscillatoriacea [62–64] and Soft-Bodied Stream Algae of California (http://dbmuseblade.colorado.edu/DiatomTwo/sbsac_site/). *Beggiatoa* [65–70]. *Spirogyra* (Soft-Bodied Stream Algae of California). Individual measurements are in Supporting Information in the file S1 Data.

The 58–175 μm wide *Rafatazmia* tubes are up to ca. 2 mm in length (Fig 6A); the ends appear not to be preserved. The tubes are divided by transverse septa into compartments that range from 30 μm to 165 μm in length. The positions of the septa are usually seen on the outside as annular grooves (Figs 2, 3D, 4, 5 and 6B). The external annulation may be visible even when the internal preservation is too poor to show any remnants of septa (Fig 3D–3F). Although the length can vary significantly between adjacent compartments (Figs 3J–3L, 4B and 4F), there is a general isometric trend in which the width of the filaments is linearly correlated with the length of the cells (Fig 7).

**Fig 8:**
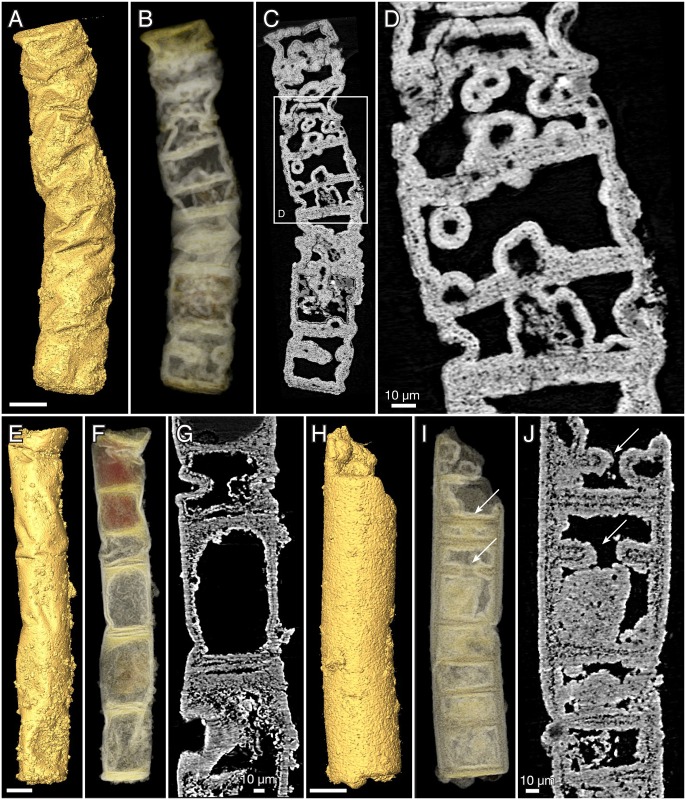
*Rafatazmia chitrakootensis* n. gen., n. sp., SRXTM renderings. (A–D) NRM X4246, surface, volume, slice. (E–G) NRM X4240, surface, volume, slice. (H–J) NRM X4242, surface, volume, slice; arrows point to new septa with central pore. Scale bars 50 μm except where otherwise noted.

Most compartments are empty, meaning that they are not filled with apatite but with secondary carbonate that was mostly dissolved during acid extraction of the fossils (see remaining carbonate crystals in Figs 2C, 2D, 4F, 4G, 5Q and 5R). A few have an irregular content of void-filling phosphate that envelopes undefined objects or fills the compartment completely, but most of these structures do not show any recurring pattern and are not deemed to be significant in terms of original morphology of biological features (Figs 4J and 8).

The septa between compartments are typically flat but sometimes bulge into adjacent compartments to form concavo-convex partitions (Figs 4B, 4C, 4F, 4G, 5Q and 5R). Some irregularities in the septa can be explained as the result of taphonomic collapse of the tube structure (Fig 8A–8D), but generally, the septa also remain undisturbed when the outer tube wall is strongly distorted (Figs 4J, 8D and 8G). The specimen in Fig 4E–4G has a general pattern of 125–145 μm distance between septa, which is broken by a single septum in the middle of one compartment (Fig 4G, arrow). This septum has a pore in the central part and possibly represents a new dividing wall under formation. A similar phenomenon is seen in Fig 8I and 8J, where two incomplete septa (arrows in J) partition larger (80–110 μm long) compartments, though not into equal parts. A more irregular pattern of incomplete septa can be seen in Fig 3K and 3L, where four consecutive septa are open in or near the middle. The irregular appearance of minute septum-like projections from the outer wall within the same region of the tube (Fig 3L, is) complicates the picture, however, emphasizing the difficulties of recognizing original structure through a taphonomic/diagenetic overprint.

The best-preserved specimens of *Rafatazmia* are shown in Fig 5. They are preserved in microgranular apatite with little addition of void-filling matter. The holotype of *R*. *chitrakootensis* (NRM X4258, Fig 5A–5L) has six compartments, separated by septa that are internally complete and externally visible as distinct furrows between the slightly convex walls of the compartments. Two of the compartments are distorted by collapse, but all have a distinct and consistent internal structure. This consists of a cylindrical body attached to the outer wall and extending toward the middle of the compartment, suspending a flattened-rhomboidal object in the centre. This object is 50–60 μm diagonally (except one 93 μm wide, Fig 5K) and 20 μm thick and is clearly visible in five of the six compartments (Fig 5F–5L). The flattened plane is aligned with the length axis of the filament. The supporting body is detached from all but the peripheral parts of the dividing septum, forming a thin planoconvex space by the septum. In the middle, where the rhomboidal object is suspended, there is a lenticular space (Fig 5C, 5F, 5G, 5I and 5K).

**Fig 9:**
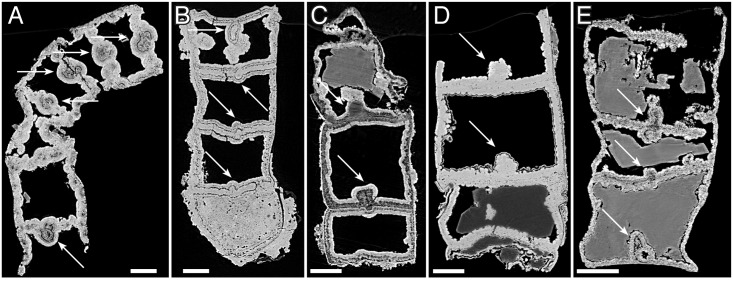
*Rafatazmia chitrakootensis* n. gen., n. sp., SRXTM virtual slices. Arrows point to diagenetic overgrowths indicating the presence of irregularities consistently placed at the centre of septa and interpreted as possible pit plugs. (A) NRM X5573. (B) NRM X5645. (C) NRM X5626. (D) NRM X5610. (E) NRM X5613. Scale bars 50 μm.

The two other specimens in Fig 5 (Fig 5M–5R) are not as completely preserved as the holotype. There is no cylindrical internal body, but most septa have a flattened or globular object attached to the central part, in some cases expressed on both sides of the septum (Fig 5R, top). In at least one case (Fig 5M–5O), their size and morphology suggest that they represent degraded specimens of the rhomboidal objects that have lost their suspending material and attached to the septal membrane. This may, however, not explain the several cases in which there are small objects consistently situated on or through the centre of septa, where they typically nucleate diagenetic spherules (Figs 4E–4G, 5P–5R and 9).

The original wall in most cases appears to have been very thin (black line at “sw” arrow in Fig 3L). In the specimen in Fig 4A–4D, the boundary representing the wall (sw) is considerably less distinct than that between the primary and secondary diagenetic overgrowth (db). A dividing gap of 2–3 μm in a polished transverse section may represent the original wall between outward- and inward-growing apatite (Fig 2C, arrow). Exceptionally, a thicker wall (about 7 μm) composed of homogenous apatite surrounds a compartment (Fig 4D, tw); this feature is interpreted to represent a modified cell with a thickened cell wall.

#### Morphology, taphonomy and diagenesis of *Denaricion* tubes

**Fig 10:**
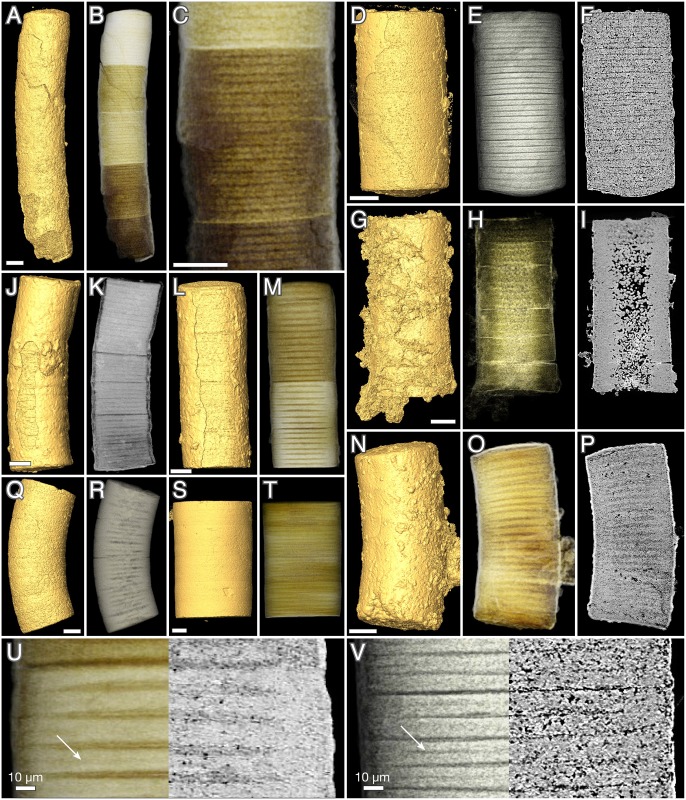
*Denaricion mendax* n. gen., n. sp., SRXTM renderings. (A–C) Holotype, NRM X5644, surface, volume, slice. (D–F, V) NRM X5604, surface, volume, slice, volume/slice. (G–I) NRM X5634, surface, volume, slice. (J, K) NRM X4233, surface, volume. (L, M, U) NRM X4234, surface, volume, volume/slice. (N–P) NRM X4244, surface, volume, slice. (Q, R) NRM X4256, surface, volume. (S–T) NRM X4235, surface, volume. Arrows in U and V point to dark lines suggesting incipient septation. Scale bars 50 μm except where otherwise noted.

The 130–275 μm wide tubes of *Denaricion* (Fig 10) are always broken at both ends, usually at planes of weakness producing flat surfaces of breakage. The longest fragment (Fig 10A–10C) is 755 μm in length, and the preserved stretches of tube are straight to evenly curved. The cross section is circular to slightly oval.

In contrast to *Rafatazmia*, *Denaricion* tubes have an almost solid interior, consisting of microgranular apatite that weakly expresses a structure like a stack of coins (Fig 10C, 10E, 10F, 10H, 10I, 10K, 10M, 10O, 10R, 10T, 10U and 10V). Void-filling apatite is generally absent, which suggests early replacement of biological tissue by microgranular apatite, not affected by later void-filling phosphatization such as in *Rafatazmia*. The “coin-stack” structure is faintly or not-at-all visible in virtual slices but can be seen in semitransparent volume renderings in projections perpendicular to the tube axes (cf. the left [volume rendering] and right [virtual slice] parts of Fig 10U and 10V, respectively). The “coins” appear to be in touch at the periphery but have a somewhat concavo-concave shape resulting in a lens-shaped volume of lower X-ray attenuation (dark) between the “coins.” Where the structure is distinctly expressed, there is a thin line of low attenuation in the middle of each “coin” (Fig 10U and 10V, arrows).

**Fig 11:**
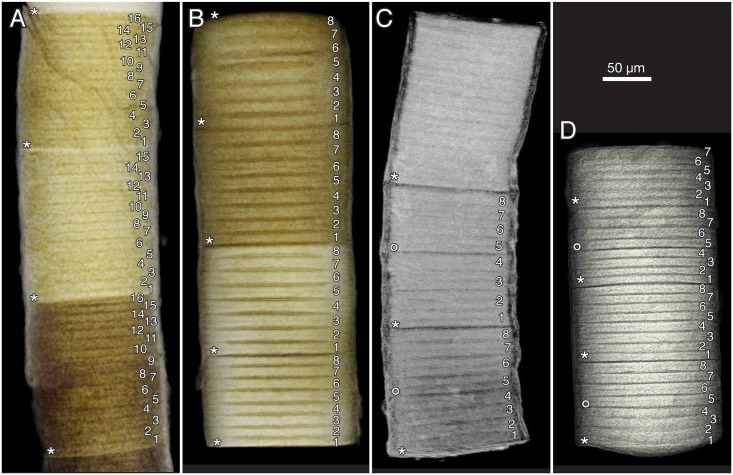
*Denaricion mendax* n. gen., n. sp., blowups of SRXTM volume renderings from Fig 10 to show 4/8/16 pattern of dividing cells within compartments. Asterisks show divisions between 8-cell compartments; rings mark weaker divisions between the fourth and the fifth cell within some compartments. (A) Same as in Fig 10B. (B) Same as in Fig 10M. (C) Same as in Fig 10K. (D) Same as in Fig 10E.

In addition to the fine stacking pattern, there is a compartmentalization expressed as a more distinct plane cutting from edge to edge (Fig 11, asterisks); this is also a plane of weakness where the tubes are usually broken off. In several cases, the planes delimit sections of different degrees of mineralization, as apparent through differences in colour and/or X-ray attenuation (e.g., Fig 10B and 10M). With minor exceptions, the number of “coins” within one such compartment is 8 or 16 (Fig 11); some compartments also have a somewhat less distinct plane between the fourth and fifth “coin” in a sequence (Fig 11C and 11D, rings), suggesting a general geometric pattern of 4/8/16.

The outer surface of the tubes is normally smooth, though it may be obscured by a diagenetic coating of apatite (Fig 10A, 10D, 10G, 10J, 10L and 10N). Occasionally, the planes of compartmentalization are visible as faint annulations beneath the coating (Fig 10L).

#### Interpretation of the tubular fossils

Filamentous organisms are widespread among microbial taxa. The first question to address is whether the tubular fossils described herein represent pro- or eukaryotes.

Size often takes a central position in the identification of fossil eukaryotes, but it has long been recognized that prokaryotic and eukaryotic cell sizes overlap considerably and that size cannot be used as an absolute criterion in fossil interpretation [71]. Bacterial cells, although typically of micrometre size, can attain dimensions of hundreds of micrometres [63]: cells of the sulfur bacterium *Thiomargarita* may be as large as 750 μm in diameter [72], and the filamentous sulfur bacterium *Beggiatoa* reaches 190 μm in width [65].

The compartments in *Rafatazmia* can confidently be identified as concatenated cells of a filamentous organism. The 4/8/16 pattern of distribution of the *Denaricion* “coins” shows them to be formed by duplication through coordinated septation, providing strong support for their interpretation as cells. Fig 7 shows a comparative diagram of filament width and cell volume in the two Vindhyan taxa versus modern oscillatoriacean cyanobacteria (*Oscillatoria* and *Lyngbya*), sulfur-oxidizing filamentous bacteria (*Beggiatoa*), and zygnematalean filamentous green algae (*Spirogyra*). The Vindhyan taxa both cluster at the upper right end of the diagram. Conspicuously, there is no overlap with the filamentous cyanobacteria, which have a filament width not exceeding 80 μm [63,64] and a maximum cell volume almost four times smaller than that of the smallest Vindhyan form. The sulfur-oxidizing *Beggiatoa* follows the same trajectory as the cyanobacteria but reaches a considerably wider diameter, about 190 μm, and in its upper range it clusters together with *Denaricion*, which, however, reaches a diameter of 275 μm. The zygnematalean *Spirogyra* in its upper size range follows the same trajectory as *Rafatazmia*. Throughout its size range, and particularly in the lower part of the range, *Spirogyra* exhibits a considerably larger cell volume than the prokaryotic filaments of similar width.

These size comparisons show that *Rafatazmia* conforms to a eukaryotic cell size distribution, with an approximately isometric size increase. *Denaricion*, on the other hand, follows a trajectory of filamentous prokaryotes, characterized by more severe constraints on cytoplasmic volume. In terms of filament width, it exceeds *Rafatazmia* and known prokaryote filamentous forms, but the tendency of the *Denaricion* cells to become thinner and form “coin stacks” with increasing filament diameter is characteristic of large filamentous cyanobacteria [64].

Yet, as *Denaricion* clearly falls outside the size range of known cyanobacteria (Fig 7; [63,64]), comparisons may rather be made with the large sulfur bacteria, in particular, *Beggiatoa*. The constraints on prokaryote cell volume are thought to be due to the limits of diffusion speed within a cytoplasm that lacks membrane-bound cytoskeletal transport mechanisms. The extreme sizes of *Beggiatoa* and some other sulfur bacteria do not reflect cytoplasmal volume, however, but rather the presence of intracellular vacuoles; the cytoplasm typically occupies only a thin veneer between the cell membrane and the vacuole [65], perhaps amounting to some 2% of the total cell volume [63]. The vacuoles of sulfur bacteria, commonly a storage site for nitrate, are prone to rapid collapse during degradation, as has been confirmed by decay experiments in the laboratory [73]. The apparent stiffness of the *Denaricion* filaments and the solid structure of its fossilized cell contents speak strongly against the cells being occupied by a liquid vacuole. Furthermore, there is no evidence in *Denaricion* of the sulfur granules that would be expected in the cytoplasm of a sulfur-oxidizing bacterium [72]. The systematic placement of *Denaricion* is thus problematic. Its living possible analogues among prokaryotes either are too small (filamentous cyanobacteria) or lack the preservation potential (sulfur-oxidizing giant filamentous bacteria) to be likely relatives. While acknowledging the possibility of extinct prokaryote branches with larger cytoplasmic volumes than in known forms, we leave the question of the affinities of *Denaricion* open at present.

A eukaryotic affinity of *Rafatazmia* is suggested primarily by its cell volume (Fig 7) and by the presence of large organelle-like objects inside the cells (Fig 5A–5L). Subcellular structures in microbial fossils are notoriously difficult to interpret, and much literature has been devoted to controversies about the nature of globular or irregular objects seen within Proterozoic microfossils [50,71,74–82]. The gist of the controversies has been whether such objects are taphonomic/diagenetic features, such as degraded and shrunken protoplasts, or whether they can be interpreted as more distinctly defined subcellular objects, in particular, nuclei or pyrenoids. A middle road was recently taken by Pang et al. [82], who suggested that globular objects in acritarchs of the late Palaeoproterozoic Ruyang biota in northern China represent shrunken protoplasts formed by the organism during encystment.

Without morphological or structural data in support, the interpretation of intracellular structures in fossils is bound to be controversial. Numerous identifications of fossilized nuclei have been made, but most of them have not withstood scrutiny, although it is known from Phanerozoic occurrences that cell nuclei, even chromosomes, can be faithfully replicated by fossilization [83]. Nuclei have been identified in Neoproterozoic embryo-like fossils based on features such as recurrence, position, shape, volumetric relationships, and evidence of closed mitosis [78,79,81], but without support from such morphological evidence, the nature of alleged fossilized nuclei is bound to be controversial.

The intracellular features of *Rafatazmia* cells are distinct, involving a rhomboidal disc suspended in the middle of each cell by a regular framework of cytoplasmic matter. Growth seemingly took place by septation of cells, and the organism occasionally produced thick-walled cells, maybe as part of an encystment process. The central object is preserved in early-diagenetic fine-grained apatite, and the recurring features of rhomboidal shape, central position, and orientation with its major plane within the length axis of the filament strongly indicate that original biological morphology is represented. Given the size of these intracellular objects, an interpretation as eukaryotic organelles is reasonable. Bacterial organelles, such as carboxysomes, are enclosed within a proteinaceous shell in microcompartments that typically are only a few hundreds of nanometres in diameter [84]; the rhomboidal bodies of *Rafatazmia* are more than a hundred times wider. Within a eukaryotic frame of reference, the *Rafatazmia* rhomboids may represent, e.g., nuclei, plastids, or pyrenoids. Given the polygonal (rhomboidal) shape, a pyrenoid is the more likely alternative.

Pyrenoids are dense proteinaceous bodies associated with chloroplasts in most groups of algae [85,86]. They are commonly involved in the production of starch—or the floridean starch of red algae—and tend to take on a polygonal shape [87]. The relation between polygonal shape and the surrounding starch plates is most commonly observed in green algae, chlorophytes [77,88], but even though floridean starch is stored in the cytoplasm rather than in the chloroplast, polygonal pyrenoids are also known from red algae, rhodophytes (Figure 2A, 2B in [89]). The number of pyrenoids in a cell or in a chloroplast varies enormously between taxa, but basal rhodophyte lineages [90–92] are generally characterized by cells having a single, centrally located plastid with a conspicuous pyrenoid [88,93], as exemplified by filamentous *Porphyridium* [94], *Chroodactylon* [95,96], and *Bangia* [97]. Later lineages tend to lose the encasing starch shells [98] and thus their angular pyrenoids.

Pyrenoids are of different sizes, but most are much smaller than the rhomboidal bodies in *Rafatazmia*. The size of a pyrenoid, however, is under comparable conditions correlated with cell size [99], and the relative sizes of the *Rafatazmia* internal bodies versus cells are fully comparable to those seen in modern algae with singular pyrenoids (Figure 4 in [97]).

Filament endings are not preserved in the fossilized *Rafatazmia*, but the evidence of incomplete septa forming within individual cells (Fig 4G, arrows, and Fig 8I and 8J) suggests that growth took place by diffusely distributed septation rather than from apex tips or specialized meristems. In rhodophytes, diffuse growth characterizes the early branching lineages, such as the bangiophytes, whereas filamentous florideophyceans have apical growth meristems [88]. The presence of a central pore in *Rafatazmia* septa and of small bodies in corresponding positions in other septa is reminiscent of the mode of septation in rhodophytes, in which new septa are commonly incomplete and the central pore may later be filled with a tubular membrane called a primary pit plug, connecting the two daughter cells; pit plugs are unique to multicellular red algae and are particularly common in the Bangiophycea/Florideophycea clade [92,100]. The preservation of the *Rafatazmia* structures in diagenetic apatite, however, does not allow any definitive comparison with rhodophyte pit plugs.

Cells with single plastids having a large pyrenoid occur in other filamentous algae, such as the chlorophytes *Klebsormidium* and *Uronema*, but these forms have parietal chloroplasts, resulting in a nonaxial position of the pyrenoid. A few zygnematalean chlorophytes (the group including *Spirogyra*, which was used for morphological comparisons in Fig 7), such as *Zygnema*, have a single pyrenoid per plastid but two plastids per cell.

The presence of a single pyrenoid per cell in *Rafatazmia* is therefore not an unambiguous character to distinguish rhodophyte versus chlorophyte affinity. The indications of pit connections and pit plugs, if corroborated, may place *Rafatazmia* within the crown-group Rhodophyta. The diffuse growth would further place it within the Bangiophycea, but the possibility that diffuse growth of filaments is a plesiomorphy for the rhodophytes makes this assignment also uncertain.

### Lobate thalli

The fabric of cyanobacteria-dominated biofilms in the stromatolitic columns contains embedded lobate thalli with cellular preservation. These differ significantly in size, structure, and preservation from the surrounding microbial communities and are analysed here as potential eukaryotes, *Ramathallus lobatus* n. gen., n. sp.

#### Taphonomy and diagenesis of *Ramathallus* thalli

**Fig 12:**
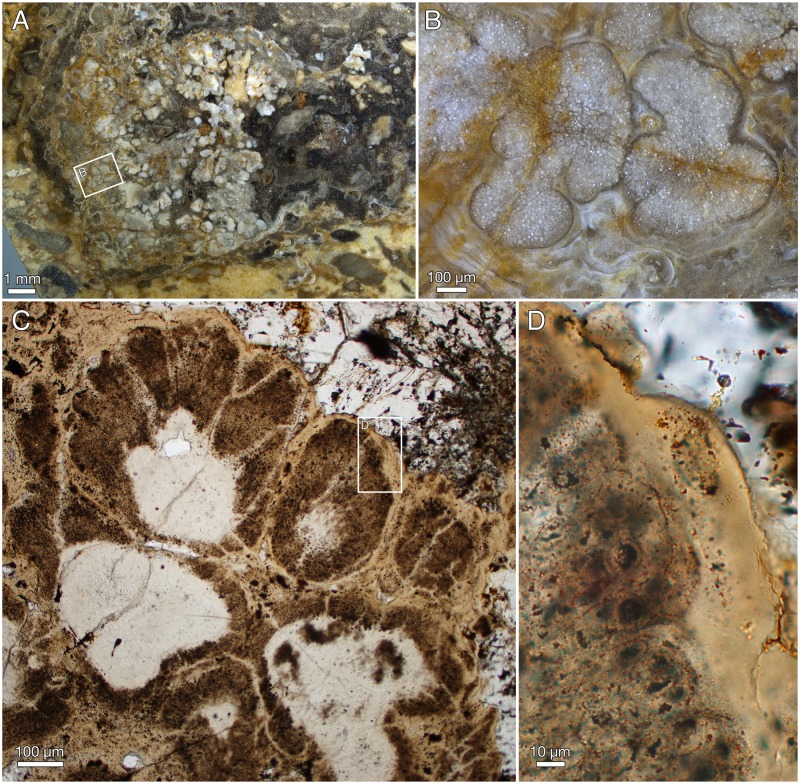
*Ramathallus lobatus* n. gen., n. sp. (A, B) NRM X5639, reflected light. (C, D) NRM X5643, thin section.

**Fig 13:**
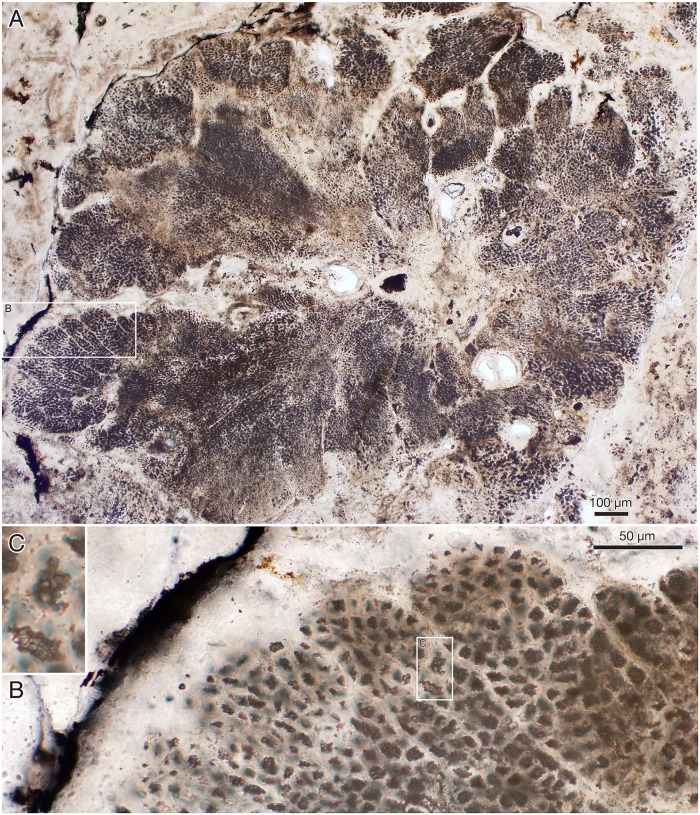
*Ramathallus lobatus* n. gen., n. sp., holotype, NRM X5638. Thin section. (A) Overview of specimen. (B) Detail of A to show cell structure in finger-like protrusions and noncellular apatitic coating. (C) Detail of B to show dark granular material within cells.

**Fig 14:**
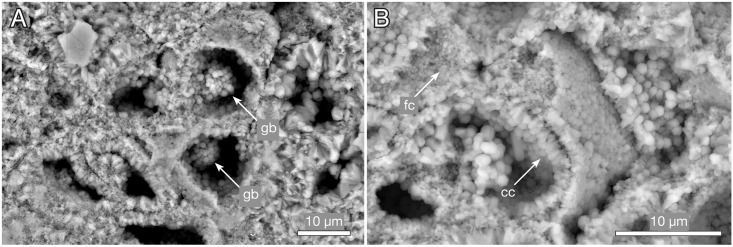
*Ramathallus lobatus* n. gen., n. sp., NRM X5640. Environmental scanning electron microscopy (ESEM) images, backscatter mode. (A) Globular bodies (gb) within cells. (B) Coarse crystals (cc) and fine crystals (fc) in diagenetically phosphatized cell walls.

The millimetre-sized lobate thalli are part of the dense apatitic stromatolitic fabric and have only been observed in sections and in untreated stromatolite slabs, not in acid-isolated residues. They appear light grey in sections under reflective light (Fig 12A), the cell lacunes being light grey or white and the walls between the cells a darker grey (Fig 12B). Under transmitted light, the 1–3 μm thick cell walls are light beige-pink with a central refractive or dark line (Fig 13B and 13C). Cell interiors are an opaque brown to black, often with a granular appearance (Fig 13C). Under the scanning electron microscope, the walls are seen as a 0.2–0.5 μm thin microcrystalline sheet coated on both sides by a more coarsely crystalline layer (e.g., Fig 14). This suggests that the original wall was considerably thinner than the 1–3 μm thick walls seen under the optical microscope, most of the thickness representing diagenetic coating. The presumed original cell walls consist of equant, approximately 0.2–0.5 μm, apatite crystals (Fig 14B, fc). The larger crystals coating the walls are dominantly hexagonal apatite tablets and prisms, 0.5–1 μm in cross-section and up to 3 μm in length (Fig 14B, cc).

**Fig 15:**
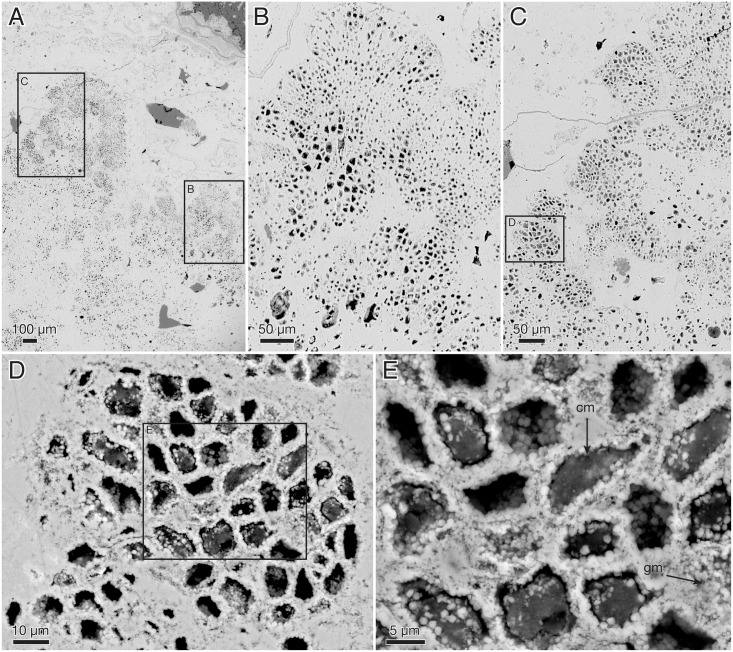
*Ramathallus lobatus* n. gen., n. sp., NRM X5638. ESEM images, backscatter mode. (A) Overview. (B and C) Details of A to show pseudoparenchymatous structure with “cell fountains” and varying cell sizes. (D and E) Details of C to show preservation of cells. Legend: cm, carbonaceous matter; gm, granular matter.

**Fig 16:**
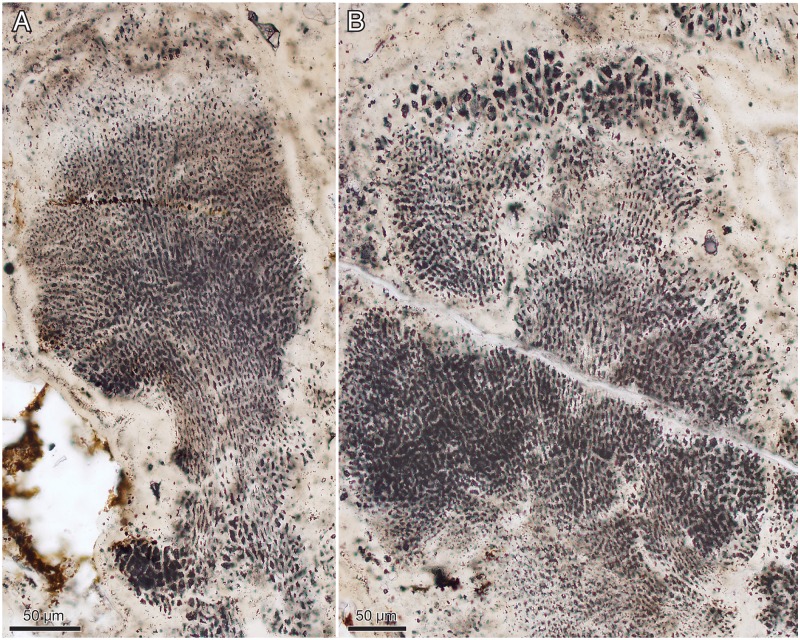
*Ramathallus lobatus* n. gen., n. sp., NRM X5638. Thin section. (A) Thallus with well-developed “cell fountains” in club-like distal part. (B) Thallus with “cell fountains” and cortex-like structure of enlarged cells.

**Fig 17:**
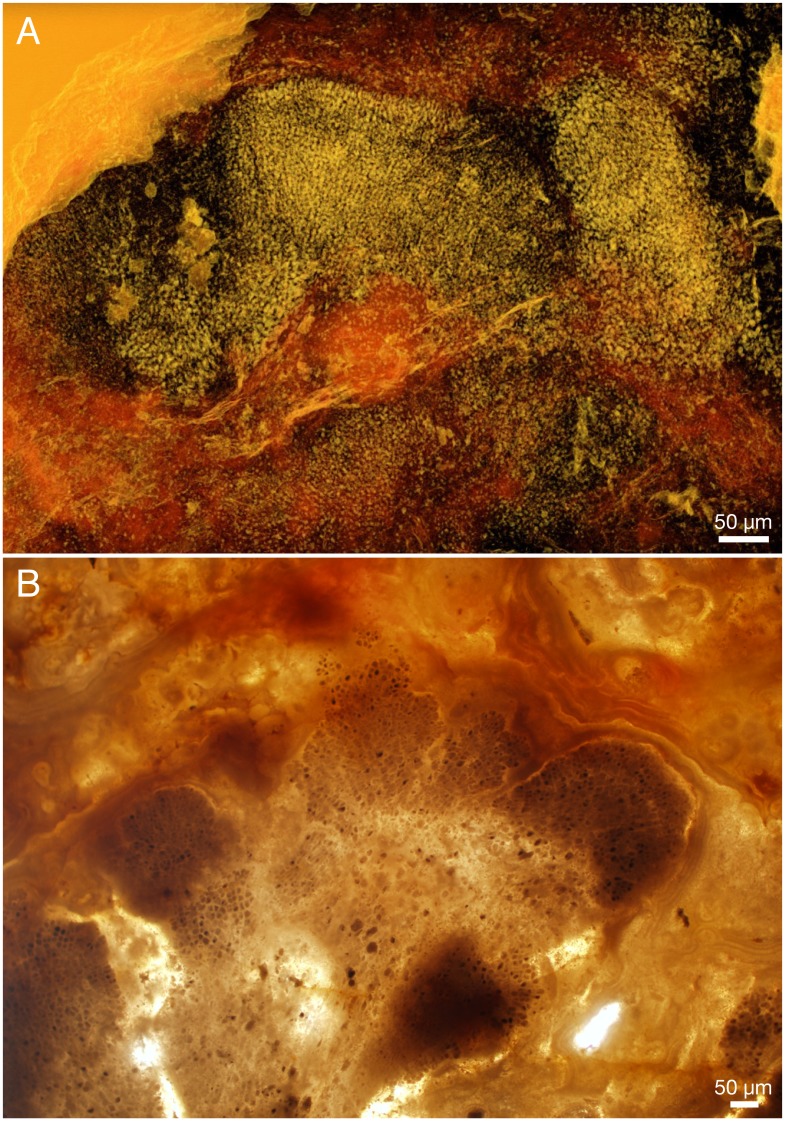
*Ramathallus lobatus* n. gen., n. sp. (A) NRM X5641, SRXTM volume rendering. (B) NRM X5642, thick section.

**Fig 18:**
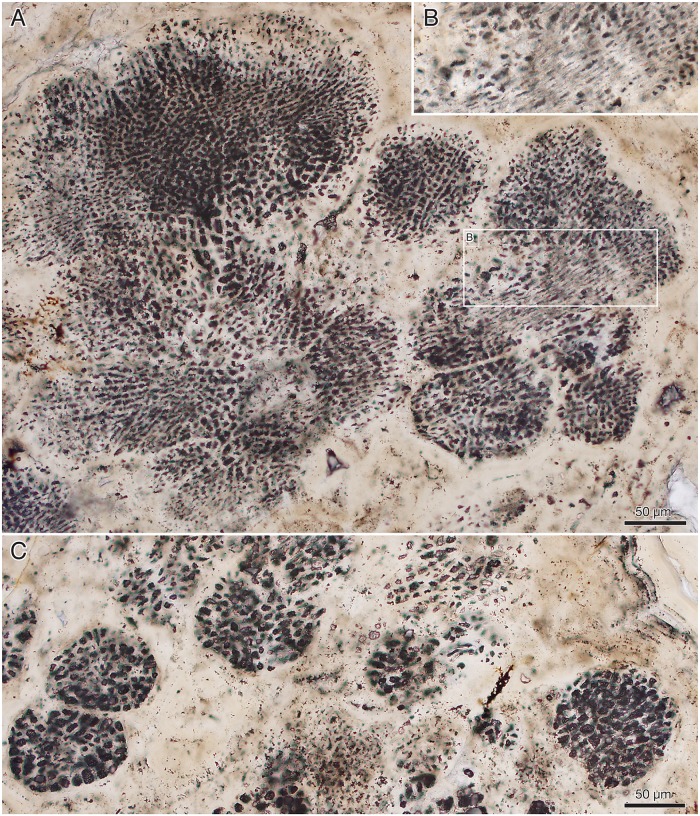
*Ramathallus lobatus* n. gen., n. sp., NRM X5638. Thin section. (A) Overview of complex thallus with cell fountains. (B) Detail of A to show elongated cells. (C) Different area to A of the same thin section, showing globular or finger-shaped protrusions.

Nondiagenetic differences in cell size are common (e.g., Fig 15B and 15C), but the exact size of a cell is difficult to judge because of diagenetic overprint. The content of the volume inside the cell walls and the degree of apatite encrustation vary, even between adjacent cells (Fig 15D and 15E). Some cells are hollow, and some are more-or-less completely filled with apatite crystals (Fig 15E, gm), but the majority contain residual carbonaceous matter with embedded apatite crystals (Fig 15E, cm), corresponding to the dark granular cell interiors seen under the light microscope (Fig 13C). Zones of stronger phosphatization within a thallus may appear as compact material with only sporadically visible cell interiors (light horizontal zone of upper part in Fig 16A); such fabric may be difficult to separate from noncellular features such as the light apatitic crust covering many thalli (e.g., Fig 12D, right, and Fig 16A, left). The latter is up to 40 μm thick and lacks the botryoidal fabric typical of void-filling apatite (cf. the diagenetic coating in *Rafatazmia*; Fig 2C and 2D). It shows undulating extinction, however, and thus implies a diagenetic overprint that may be coeval with the void-filling apatite, which frequently overlies it (see Figs 17B and 18C, upper right). The morphology and distribution suggest that it replaces an original biological feature of the fossils.

#### Morphology of *Ramathallus* thalli

The thalli are megascopic, ranging from approximately half a millimetre up to well over 3 mm across. Aggregations of thalli can attain a size of more than a centimetre (Fig 12A). The thalli are lobate in shape and typically project bulbous extensions (Figs 12, 13, 17 and 18) radiating from a central point of the structure (Fig 13A). Protrusions may take the form of separate globular or finger-shaped objects or be more closely adpressed to each other, separated by deep invaginations (Figs 12C, 13B and 18C). Some protrusions have a narrower stalk-like base and a more bulbous distal part (Fig 16A). The size of single cells ranges from approximately 5 μm to 15 μm. In the projecting parts of the thallus, the cells are lined up in “fountains,” producing a pattern typical of pseudoparenchymatous tissue (Figs 15B and 16–18). These cells are typically elongated in the direction of the cell row, and in the narrower regions of such a protrusion, the cell rows take on a stretched-out, filamentous appearance (Fig 18B).

**Fig 19:**
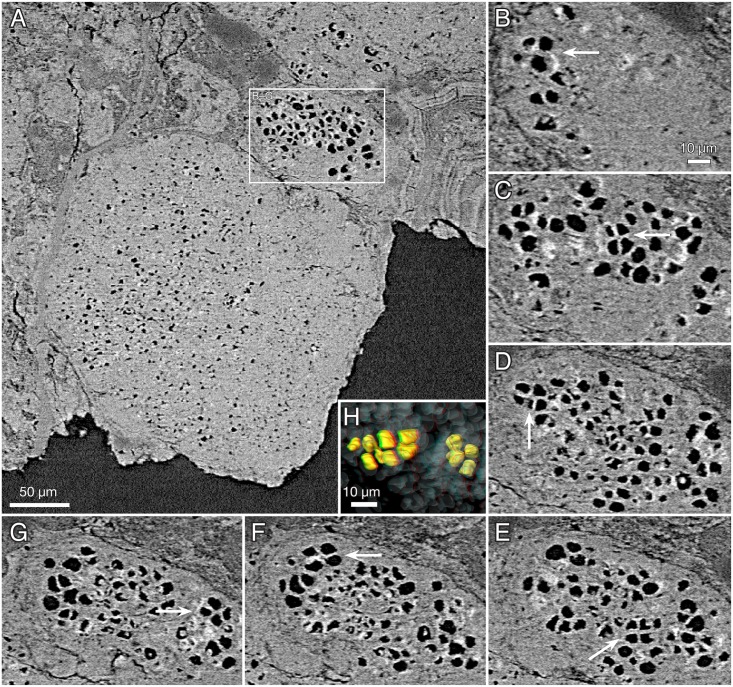
*Ramathallus lobatus* n. gen., n. sp., NRM X5641. SRXTM slices. (A) Larger field of view to show the location of the imaged volume. (B–G) Consecutive sections through volume of larger cells, where putative tetraspores are marked with arrows. (H) Stereo anaglyph showing surface-rendered cell volumes of three putative tetraspores (yellow) surrounded by nontetradially arranged cells (grey, transparent).

The smallest cells are usually in the “cell fountains,” whereas larger ones may appear in the bulbous upper parts of a protrusion (Figs 12D and 16B), in globular bodies appearing near the base of larger protrusions (Fig 16A, lower left), or as separate smaller protrusions (Figs 15A, 15C–15E, 18C and 19). The larger-celled globular bodies often do not have the same pseudoparenchymatous pattern as the larger protrusions but are more irregularly packed. In parts of the smaller bodies, the cells are repeatedly arranged as an isolated quadruplet lying in one plane, forming a four-leaf-clover pattern (Fig 19B–19G, arrows, Fig 19H). Larger cells commonly have a small internal globular body, coated with apatite crystals similar to those lining the inside of the cell walls (Fig 14A, gb)

Surrounding most of the thalli is an up to 40 μm thick homogenous apatitic coating (Figs 12C, 12D, 13A, 13B, 16A, 18A and 18C). It is continuous with, and identical in structure and colour with, the sheaths separating adjacent thallus protrusions (Figs 12C and 13B) and shows no evidence of a cellular organization. A similar coating also lines lacunae that are sometimes found in the interior of the complex thalli (Fig 13A; cf. also Figs 12C, 15A and 17B). The presence of a diagenetic layered overgrowth makes it sometimes difficult to determine the presence and extension of the homogenous outer layer (Figs 12B, 16A, 17B and 18C, upper right).

#### Interpretation of *Ramathallus*

*Ramathallus* was a sessile benthic organism, as indicated by its growth mode, with lobate protrusions radiating from a centre, and its in situ preservation within the Vindhyan microbial mats. The lobes were mainly composed of pseudoparenchymatous tissue in the form of “cell fountains.” Cell sizes were highly variable. Apparent tetraspores (the quadruplets in Fig 19) were present in localized places in the smaller globular protrusions. The thallus was embedded in a noncellular matrix that also penetrated between the lobes. No distinct cellular cortex has been observed, but the expanded cells at the outer portions of the thallus underneath the noncellular coat (Figs 12D and 16B) may have been specialized as meristematic cells and thereby represent apical growth.

Pseudoparenchymatous tissue formation is a basic way of making a resilient thallus through the coordinated growth of coalescing filaments. This type of structure is widespread among organisms with fundamentally filamentous growth, and as such, it is prone to convergence. It is characteristic of fungi and algae, particularly florideophycean rhodophytes, as well as some phaeophytes and chlorophytes [88]. Certain chroococcalean cyanobacteria, such as *Chloroglea* and *Entophysalis*, form three-dimensional multilayered colonies in which cells may be lined up in a pseudoparenchymatous pattern, but they tend to be irregularly arranged [101], not showing the distinct “cell fountains” of eukaryotic pseudoparenchyma. Nor do they display the complex and fleshy thallus characteristic of many eukaryotic algae as well as *Ramathallus*.

*Ramathallus* resembles the similarly preserved and similarly sized algal fossils in the Ediacaran Doushantuo phosphorites of China—in particular, the pseudoparenchymatous, lobate taxa *Thallophyca* Zhang (1989 [102]), *Thallophycoides* (Zhang and Yuan (1992 [103]), *Paramecia* Zhang and Yuan (1992 [103]), and *Gremiphyca* (Zhang et al. (1998 [104]). These taxa were reinvestigated by Xiao et al. [105], who compared them mutually and with fossil and modern algae on the basis of thallus morphology, cellular architecture, mode of growth, and putative reproductive structures. The authors concluded tentatively that *Thallophycoides* and *Gremiphyca* are likely stem-group florideophyceans, whereas *Paramecia* and *Thallophyca* are stem-group corallines and consequently belong within the crown-group florideophyceans, [105].

*Thallophycoides* and *Gremiphyca* have globular to nodular thalli with no evidence of attachment. The pseudoparenchymatous structure is simple, with uniform cell size and no cortex–medulla differentiation. The spherical thalli of *Wengania* Zhang (1989 [102]) may represent early developmental stages of these simple forms [105].

*Thallophyca* has a foliose or lobate thallus with conspicuous pseudoparenchyma built up of “cell fountains,” probable tetraspores, and a distinct cellular cortex with cells smaller than those of the medulla and oriented parallel or perpendicular to the surface of the thallus. In addition, it has clusters of larger cells—“cell islands”—embedded in the tissue, sphaeroidal cavities, cylindrical invaginations, and sorus-like structures that have all been interpreted as possible reproductive features [105]. *Paramecia* has a nodular, compartmentalized thallus with less-conspicuous pseudoparenchyma than *Thallophyca* and a distinct cortex–medulla differentiation; it too has embedded “cell islands” and cavities that may be involved in reproduction [105].

The Vindhyan *Ramathallus* differs from *Thallophycoides* and *Gremiphyca* in its more complex, lobate thallus, nonuniform cell size, distinct “cell fountains,” and a cortex-like region of enlarged apical cells. It is most similar to *Thallophyca* in its complex thallus and conspicuous “cell fountains” but differs from both *Thallophyca* and *Paramecia* in lacking a distinct cortex, “cell islands,” and cavities of possible reproductive nature (the cavities seen in Figs 12C and 13A may be of diagenetic origin). The presence of tetraspore-like structures in both *Ramathallus* and *Thallophyca* may possibly signify a common feature, but their occurrence in the respective taxa is not consistent. In *Ramathallus*, the structures occur scattered among cells of comparable size in large-celled globules, whereas in *Tetrathallus* they occur as large-celled aggregates within masses of considerably smaller cells. In neither case is the biological significance of the structures clear.

*Ramathallus* is thus taxonomically distinct from the billion-year younger Doushantuo forms, but its lobate thalli with distinct pseudoparenchyma, “cell fountains,” apical growth, and cortex-like modifications of the distal parts of the lobes all suggest affinity to the Doushantuo taxa; comparisons can be made with *Thallophyca* in particular. As discussed by Xiao et al. [105], the listed characters are most consistent with florideophycean rhodophytes, although like *Ramathallus* the Doushantuo taxa lack evidence of pit connections, a common feature among florideophyceans. Based on the vegetative characters of *Ramathallus*, in the absence of clear reproductive features in the thallus, we tentatively propose a stem-group florideophycean affinity of the Vindhyan taxon, which would place them within the crown-group Rhodophyta.

### Geological age

Given the remarkably old age that we cite for the rhodophyte-like fossils reported herein, it is necessary to discuss in some detail the reliability of the ~1.6 Ga date for the Tirohan Dolomite. Vindhyan stratigraphy has generated a considerable amount of discussion and controversy over the years, and even in the recent literature, age estimates for the Semri Group (Lower Vindhyan) are given as either Palaeo–Mesoproterozoic, as indicated by geochronology [106,107], or Ediacaran–Cambrian, as suggested with reference to fossil content [108,109].

Correlation between the Chitrakoot Formation and the Semri sequence in the Son Valley is not straightforward [110], but regional isopach and lithofacies interpretations suggest that the Chitrakoot Formation belongs within the upper part of the Semri Group; in particular, correlation is suggested between the Tirohan Dolomite at Chitrakoot and the Rohtas Limestone (Rohtasgahr Limestone of some authors) in the Son Valley [111].

**Fig 20:**
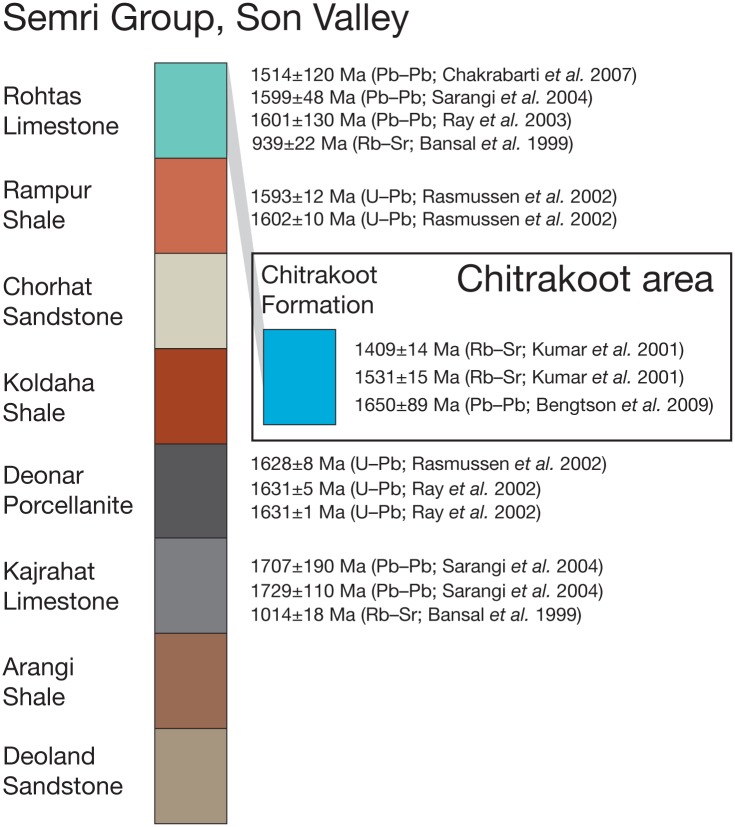
Summary of recent published results from radiometric age determinations of Lower Vindhyan rocks in the Son Valley and the Chitrakoot area. The shaded band indicates correlation of the Tirohan Dolomite (Chitrakoot Formation) with the Rohtas Limestone. Uncertainties are 2σ.

Recent geochronological results from the Semri Group in the region are summarized in Fig 20. The Rohtas Limestone in Son Valley has been dated by Pb–Pb isochrons to 1,601±130 Ma [110], 1,599±48 Ma [112], and 1,514±120 Ma [113], respectively. These dates are in agreement with the more precise U–Pb dates of 1,602±10 Ma and 1,593±12 Ma obtained from euhedral magmatic zircon grains in tuffs from the immediately underlying Rampur Shale [114]. Further down in the Son Valley sequence, tuff layers in the Deonar Porcellanite have been dated with the same zircon U–Pb method by two independent laboratories to 1,631±5 Ma, 1,631±1 Ma [115], and 1,628±8 Ma [114], respectively. Still further down, the Kajrahat Limestone has yielded two Pb–Pb isochron dates of 1,707±190 Ma and 1,729±110 Ma [112]. From the Kajrahat and Rohtas Limestones have also been published two Rb–Sr dates of 1,014±18 Ma and 939±22 Ma, respectively [116]. These anomalous dates may be affected by late illite formation [112] but in any case do not support an Ediacaran–Cambrian age of the Semri Group.

A kimberlite pipe at Majhgawan, about 30 km southwest of Chitrakoot, intrudes the Kaimur Sandstone, the lowermost unit of the Upper Vindhyan sequence, which unconformably overlies the Semri Group. The Kimberlite has yielded a mean Ar–Ar age of 1,073.5±13.7 Ma [117], confirming the pre-Neoproterozoic age of the Lower Vindhyans. For broader reviews of geochronological work on the Vindhyans, see Ray [106], Azmi et al. [108], and Basu and Bickford [107].

The presumed correlative equivalents of the Tirohan Dolomite in the Son Valley have thus been dated with precision and congruence to be very close to 1.6 Ga. This is within error margins identical to the 1,650±89 Ma age obtained from a Pb–Pb isochron dating directly of the fossiliferous phosphorite in the Tirohan Dolomite at Chitrakoot [52]. Also from the Chitrakoot area, Kumar et al. [118] obtained six Rb–Sr dates from glauconies in the Chitrakoot Formation, ranging from 1,409±14 Ma to 1,531±15 Ma. Although younger by some 100–200 million years than the above cited dates, the model Rb–Sr ages from the glauconite remain in agreement with an old, Palaeo–Mesoproterozoic age of the Lower Vindhyan. The comparatively young age obtained by Kumar et al. may be due to the well-known tendency of Palaeozoic or older glauconite to yield Rb–Sr ages that appear to be too young by 10%–20% [119], a phenomenon that can persist even through leaching experiments using a variety of agents [120], including the HCl leaching performed by Kumar et al. [118]. Ages that are apparently too young even after leaching may also indicate later recrystallization of glauconite [120], although Kumar et al. [118] consider this unlikely for their samples.

Biostratigraphic arguments have been forwarded in support of a considerably younger age, Ediacaran–Cambrian, of the Lower Vindhyans [55,108,109]. With regard to the claims of Cambrian fossils in the Tirohan Dolomite [55,56,108], these were discussed and refuted by us [52] as being based on misidentified algae (*Rafatazmia* and *Denaricion* herein), cyanobacterial biomat fragments, and gas bubbles. The only cited radiometric date that would support an Ediacaran–Cambrian age of the Son Valley Lower Vindhyans is a “preliminary” Ar–Ar date of 617±3.5 Ma from the Deonar Porcellanite Formation, published only in a conference abstract [121] and never substantiated.

A recent study of drill cores through allegedly Lower Vindhyan sedimentary rocks in the western Vindhyan sub-basin, Chambal Valley, Rajasthan, revealed a succession of diverse and well-preserved acanthomorph acritarchs of Ediacaran aspect [109]. The authors offer two alternative interpretations of this discovery: the Semri Group is Ediacaran in age or complex acanthomorph acritrarchs evolved much earlier than hitherto supposed. An additional alternative may be that the correlation of the investigated sequence with the Lower Vindhyan in Son Valley/Chitrakoot is incorrect. A major problem in Vindhyan stratigraphy is the lack of reliable correlation between the eastern (Son Valley and Chitrakoot) and western (Rajasthan) sub-basins owing to differences in lithology, lack of common marker horizons, lack of congruence in chemostratigraphy, lack of biostratigraphically useful fossils, and lack of continuous outcrops [110,122]. The Chitrakoot Formation indeed contains large acritarchs with complex wall structure [40,54], but these are not acanthomorphs and rather seem comparable to the assemblages in the ~1.7 Ga Ruyang Group in northern China [38] and the 1.5–1.4 Ga Roper Group in northern Australia [123].

The ~1.6 Ga geochronological date of the Tirohan Dolomite is robust. This conclusion is based on a congruence of radiometric measurements, but particularly important are the U–Pb analyses of magmatic zircon, the most precise and accurate absolute dating method for rocks of this age, in the Son Valley Lower Vindhyan [114,115], as well as the direct Pb–Pb isochron dating of the fossiliferous phosphorite [52]. Apparent conflicts with biostratigraphic considerations are likely to be resolved through reexamination of the fossil evidence [52], but in general they serve as an indication that the Proterozoic fossil record of both micro- and megafossils is richer than hitherto recognized. The preservation window opened by the phosphorite deposit in the Tirohan Dolomite thus underscores that we have been missing a lot of the biotic diversity in the Palaeo–Mesoproterozoic because of the dearth of lithologies suitable for fossilization of delicate tissues.

### Rhodophyte phylogeny and age

Together with chlorophytes and glaucophytes, the rhodophytes belong to the Archaeplastida [124], which seem to have derived their chloroplasts from a single endosymbiotic event involving a cyanobacterial donor [125]. Other algal groups obtained their chloroplasts from secondary endosymbiosis with different archaeoplastid groups [126]. The Vindhyan putative rhodophytes, having already diversified into multicellular filamentous and lobate forms, would have been subsequent to the primary symbiotic event, and their photosynthetic ability is suggested by the presence of pyrenoid-like structures in *Rafatazmia* and an apparent phototactic growth mode in *Ramathallus*.

Yoon et al. [127] recognized seven rhodophyte lineages, among which the florideophyceans and bangiophyceans were united as sister groups within one clade. The basal phylogenetic relationships were largely unresolved, although the unicellular acidophilic Cyanidiophyceae came out as a sister group to the remaining rhodophytes.

The presented data suggest that crown-group rhodophytes—the putative florideophycean *Ramathallus* and possible bangiophycean *Rafatazmia—*were present in the ~1.6 Ga Vindhyan stromatolitic microbialites. Whether or not these two taxa belong to the extant clades as proposed, they seem to be part of the unresolved clade that forms a sister group to the unicellular cyanidiophyceans and so would belong to crown-group rhodophytes. This runs contrary to recent concepts about the evolutionary timing of eukaryote lineages. Several recent molecular-clock studies [16,17,48] have had problems in using the ~1.2 Ga *Bangiomorpha* [46] as a calibration point for bangiophycean or even rhodophyte appearance, because it had seemed too old to make sense.

Molecular-clock estimates of lineage divergence times in the tree of life are heavily dependent on calibration based on occurrences and events in the fossil record. Uncertainties in fossil calibration have a dominant effect on the dating of nodes in molecular phylogenetic trees [128,129]. The first representative of a lineage in the fossil record postdates the origination of that lineage by an unknown interval of time; thus, the age of a fossil only gives a minimum age for the lineage. Depending on the fossilization potential of the particular organisms and the availability of suitable rocks, the interval of uncertainty can be of any length and may be more or less easy to assess. For the Phanerozoic, the richness of the rock record and the presence of easily fossilized organisms allow the construction of reasonably reliable calibration intervals [130], but the Proterozoic is by comparison unchartered territory in which fossiliferous deposits are scarce and fossils often controversial in nature. Calculated Proterozoic divergence dates are commonly based on extrapolations from Phanerozoic calibration intervals.

Berney and Pawlowski [48] based a calibration of the eukaryote tree on the Phanerozoic microfossil record of coccolithophorids, diatoms, and dinoflagellates. Using a Bayesian relaxed-clock model, they arrived at dramatically different dates for the divergence of major eukaryote groups depending on whether or not Proterozoic fossils were included in the calibration. When *Bangiomorpha* was used to set a minimum age for the red algae, the resulting age for LECA was 3,868 Ma, with a 95% confidence interval of 4,182–1,830 Ma, whereas exclusion of *Bangiomorpha* gave a LECA age of 1,126 Ma (1,357–947 Ma). For this reason, they concluded that *Bangiomorpha* and other proposed Proterozoic algal and fungal taxa more likely represent basal lineages of eukaryotes convergent on extant lineages and that calibration should be based only on Phanerozoic data.

Parfrey et al. [17] similarly compared divergence dates depending on whether or not Proterozoic fossils were used for calibration. Their relaxed-clock results were more stable to the inclusion/exclusion of Proterozoic fossils than those of Berney and Pawlowski [48], yet they found the origination date of the rhodophytes to shift considerably, from 1,285–1,180 Ma (95% credible range) to 959–625 Ma, when Proterozoic taxa were excluded. Parfrey et al. [17] favoured the inclusion of the Proterozoic fossils for calibration, leading to a LECA age of 1,866–1,679 Ma, older than that of most modern molecular-clock estimates. This result, however, required *Bangiomorpha* to be placed at the base of the crown-group rhodophytes, not with the bangiophyceans.

Sharpe et al. [16], using a relaxed-clock model, estimated the origin of rhodophytes to 996 Ma, with a 95% credible interval of 1,186–801 Ma. They found *Bangiomorpha*, treated as a stem-group multicellular rhodophyte lineage, to be at odds with the calculations and excluded it as a calibration point. They argued that unless *Bangiomorpha* was not a red alga or incorrectly dated, currently used Bayesian relaxed-clock models might be insufficient to deal with changes in evolutionary rates within lineages.

Yang et al. [47] accepted *Bangiomorpha* as a rhodophyte, though again not as a bangiophycean but as an early rhodophyte stem group, for calibration. When testing two approaches to assigning prior distributions within their Bayesian relaxed-clock model, they found that the date for bangiophycean/florideophycean divergence shifted from 943 Ma to 1,661 Ma, and because the older date seemingly contradicted the fossil evidence, they preferred the approach yielding the lower age. They also found that their results were sensitive to the inclusion of *Bangiomorpha*, but having included it, they arrived at a rhodophyte/chlorophyte divergence date of 1,693 Ma (1,925–1,484 Ma). This is older even than the LECA age of most studies.

There remain a number of theoretical problems to be resolved with molecular-clock datings of ancient divergences, but the pattern that emerges from the above examples is that current models are not adequate to deal with a minimum-age constraint for crown-group multicellular rhodophytes at 1.2 Ga, as defined by *Bangiomorpha*. In order to fit the pattern of calibrations based mainly on Phanerozoic data, *Bangiomorpha* has to be reclassified as a stem-group eukaryote, as a stem-group rhodophyte, or, at best, near the first representative of the crown-group rhodophytes.

*Rafatazmia* and *Ramathallus* are some 400 million years older than *Bangiomorpha* and are tentatively interpreted as representing multicellular crown-group rhodophytes already diversified into filamentous and fleshy forms. This would change the constraints dramatically for the timing of divergences within early rhodophytes and within Eukaryota as a whole. Maybe this can be accommodated by taking into account previously downplayed possibilities, such as changes in substitution rates in multiple lineages over time [131,132], but the basic task should be to discover and study Archaean and Proterozoic fossils and base interpretations on the available evidence rather than on how they happen to fit into currently accepted time frames [133–137].

### Systematic palaeontology

**Genus *Rafatazmia* Bengtson n. gen.**
- *Type and only species*. *Rafatazmia chitrakootensis* Bengtson n. sp.
- *Etymology*. In honour of Dr. Rafat Azmi. The name is feminine in gender.
- *Diagnosis*. Nonbranching filamentous alga, 58–175 μm in width. Cells of different sizes, with lengths from half to more than twice the width. Diffuse growth by septation. New septa with central pore; some septa also with central globular object, sometimes penetrating the septum; these structures are possible pit connections and pit plugs. Large rhomboidal disk suspended in the middle of each cell; interpreted as a pyrenoid.

***Rafatazmia chitrakootensis* Bengtson n. sp. Figs 2–6, 8 and 9.**
- *Holotype*. Swedish Museum of Natural History NRM X4258 (Fig 5A–5L).
- *Type stratum*. Tirohan Dolomite, Jankikund section, Chitrakoot, India. 25°09′46″N, 080°52′05″E.
- *Etymology*. From its occurrence at Chitrakoot.
- *Diagnosis*. As for the genus.

**Genus *Denaricion* Bengtson n. gen.**
- *Type and only species*. *Denaricion mendax* Bengtson n. sp.
- *Etymology*. From Latin *denarius*, a silver coin, and Greek *kion*, “pillar,” referring to the likeness of the internal structure to a stack of coins. The name is masculine in gender.
- *Diagnosis*. Nonbranching filamentous alga or prokaryote, 130–275 μm in width. Cells short, coin-shaped. Growth by coordinated septation producing a geometric 4/8/16 pattern of cells within compartments that are at least partly sealed from each other by a solid septum.

***Denaricion mendax* Bengtson n. sp. Figs 10 and 11.**
- *Holotype*. Swedish Museum of Natural History NRM X5644 (Fig 10A–10C).
- *Type stratum*. Tirohan Dolomite, Jankikund section, Chitrakoot, India. 25°09′32″N, 080°52′52″E.
- *Etymology*. Latin *mendax*, “deceptive,” for its enigmatic nature.
- *Diagnosis*. As for the genus.

**Genus *Ramathallus* Sallstedt n. gen.**
- *Type and only species*. *Ramathallus lobatus* Sallstedt n. sp.
- *Etymology*. From Rama and Latin *thallus*, “branch.” The name is masculine in gender.
- *Diagnosis*. Lobate thallus with globular and finger-like branches diverging from a central area. Pseudoparenchymatous tissue often forming “cell fountains.” Cell sizes variable from about 5 to 15 μm. Apparent tetraspores occur in globular bodies. Noncellular matrix up to 40 μm thick covers the thallus. No distinct cortex, but distal layer of cells in a branch are sometimes larger than those of the adjacent pseudoparenchymatous tissue.

***Ramathallus lobatus* Sallstedt, n. sp. Figs 12–19.**
- *Holotype*. Swedish Museum of Natural History NRM X5638, large specimen in Fig 13.
- *Type stratum*. Tirohan Dolomite, Jankikund section, Chitrakoot, India. 25°09′46″N, 080°52′06″E.
- *Etymology*. Latin *lobatus*, “lobate.”
- *Diagnosis*. As for the genus.

## Materials and methods

The studied material derives from field work at Jankikund in November 2006 and January 2011 and was augmented by microfossils kindly supplied by R. Azmi. To isolate phosphatized fossils from the carbonate rocks, samples were dissolved in 10% buffered acetic acid, and the residues were manually picked for microfossils. SEM/ESEM was carried out on a Hitachi S4300 Field Emission scanning electron microscope, a Philips XL 30 ESEM-FEG, and a Quanta ESEM-FEG 650 field emission microscope. Synchrotron-radiation X-ray tomographic microscopy (SRXTM) was done at the X02DA TOMCAT beamline at the Swiss Light Source, Paul Scherrer Institut, Villigen, Switzerland. Visualization was done using Avizo 9.1.1 (FEI Company). All specimens are in the collections of the Swedish Museum of Natural History. Each figured specimen has a unique museum number, stated in the respective figure caption in the article. Tomographic data are deposited in the Dryad Digital Repository: http://dx.doi.org/10.5061/dryad.gh221 [138].

### Nomenclature

The electronic version of this article in Portable Document Format (PDF) in a work with an ISSN or ISBN will represent a published work according to the *International Code of Nomenclature for algae*, *fungi*, *and plants*, and hence, the new names contained in the electronic publication of a PLOS article are effectively published under that Code from the electronic edition alone, so there is no longer any need to provide printed copies. The online version of this work is archived and available from the following digital repositories: PubMed Central, LOCKSS, and DiVA (www.diva-portal.org/).

## Supporting information

S1 Data Data for Fig 7. Measurements of filamentous taxa used to produce Fig 7. See figure text for literature sources.
(XLSX)

## Abbreviations

ESEM: environmental scanning electron microscopy
FECA: first eukaryote common ancestor
LECA: last eukaryote common ancestor
SEM: scanning electron microscopy
SRXTM: synchrotron-radiation X-ray tomographic microscopy
